# A computational study of the addition of ReO_3_L (L = Cl^−^, CH_3_, OCH_3_ and Cp) to ethenone

**DOI:** 10.1186/s40064-016-2012-0

**Published:** 2016-03-22

**Authors:** Albert Aniagyei, Richard Tia, Evans Adei

**Affiliations:** Computational and Theoretical Chemistry Laboratory, Department of Chemistry, Kwame Nkrumah University of Science and Technology, Kumasi, Ghana

**Keywords:** Ketene oxidation, Chemoselectivity, Periselectivity, Metalla-2,4-dioxolane

## Abstract

The periselectivity and chemoselectivity of the addition of transition metal oxides of the type ReO_3_L (L = Cl, CH_3_, OCH_3_ and Cp) to ethenone have been explored at the MO6 and B3LYP/LACVP* levels of theory. The activation barriers and reaction energies for the stepwise and concerted addition pathways involving multiple spin states have been computed. In the reaction of ReO_3_L (L = Cl^−^, OCH_3_, CH_3_ and Cp) with ethenone, the concerted [2 + 2] addition of the metal oxide across the C=C and C=O double bond to form either metalla-2-oxetane-3-one or metalla-2,4-dioxolane is the most kinetically favored over the formation of metalla-2,5-dioxolane-3-one from the direct [3 + 2] addition pathway. The trends in activation and reaction energies for the formation of metalla-2-oxetane-3-one and metalla-2,4-dioxolane are Cp < Cl^−^ < OCH_3_ < CH_3_ and Cp < OCH_3_ < CH_3_ < Cl^−^ and for the reaction energies are Cp < OCH_3_ < Cl^−^ < CH_3_ and Cp < CH_3_ < OCH_3_ < Cl CH_3_. The concerted [3 + 2] addition of the metal oxide across the C=C double of the ethenone to form species metalla-2,5-dioxolane-3-one is thermodynamically the most favored for the ligand L = Cp. The direct [2 + 2] addition pathways leading to the formations of metalla-2-oxetane-3-one and metalla-2,4-dioxolane is thermodynamically the most favored for the ligands L = OCH_3_ and Cl^−^. The difference between the calculated [2 + 2] activation barriers for the addition of the metal oxide LReO_3_ across the C=C and C=O functionalities of ethenone are small except for the case of L = Cl^−^ and OCH_3_. The rearrangement of the metalla-2-oxetane-3-one–metalla-2,5-dioxolane-3-one even though feasible, are unfavorable due to high activation energies of their rate-determining steps. For the rearrangement of the metalla-2-oxetane-3-one to metalla-2,5-dioxolane-3-one, the trends in activation barriers is found to follow the order OCH_3_ < Cl^−^ < CH_3_ < Cp. The trends in the activation energies for the most favorable [2 + 2] addition pathways for the LReO_3_–ethenone system is CH_3_ > CH_3_O^−^ > Cl^−^ > Cp. For the analogous ethylene–LReO_3_ system, the trends in activation and reaction energies for the most favorable [3 + 2] addition pathway is CH_3_ > CH_3_O^−^ > Cl^−^ > Cp [10]. Even though the most favored pathway in the ethylene-LReO_3_ system is the [3 + 2] addition pathway and that on the LReO_3_–ethenone is the [2 + 2] addition pathway, the trends in the activation energies for both pathways are the same, i.e. CH_3_ > CH_3_O^−^ > Cl^−^ > Cp. However, the trends in reaction energies are quite different due to different product stabilities. The formation of the acetic acid precursor through the direct addition pathways was unsuccessful for all the ligands studied. The formation of the acetic acid precursor through the cyclization of the metalla-2-oxetane-3-one is only possible for the ligands L = Cl^−^, CH_3_ whiles for the cyclization of metalla-2-oxetane-4-one to the acetic acid precursor is only possible for the ligand L = CH_3_. Although there are spin-crossover reaction observed for the ligands L = Cl^−^, CH_3_ and CH_3_O^−^, the reactions occurring on the single surfaces have been found to occur with lower energies than their spin-crossover counterparts.

## Background

Reactive intermediates are employed in increasingly diverse ways for synthetic purposes, and understanding of the underlying chemistry has become an essential part of synthesis. The development of new catalytic reactions that enhance chemical transformations with high selectivity and efficiency is a grand challenge in chemical research.

Ethenone, the simplest member of the ketene family, is a reactive intermediate with many useful synthetic applications in chemical industry. Ketenes are widely applied in synthetic chemical processes such as ketene dimers for conversion to 1,3-cyclobutanediols as replacements for bisphenol A in the preparation of polyesters and polycarbonates, manufacture of acetic acid and in vital pharmaceutical applications such as the preparation of β-lactam antibiotics (Ulrich 1967, 2009).

Experimental and theoretical studies focusing on the mechanism of addition of transition metal-oxo species of the type LReO_3_ to the C=C double bonds addition to ketene has been reported (Deubel et al. 2001; Middleditch et al. 2007; Herrmann et al. 1985, 1986, 1994).

Middleditch et al. (2007) in an experimental study of the reaction of rhenium (VII) trioxo complexes containing the ligand sets scorpionate, [HB(pz)_3_]ReO_3_, [Ph-B(pz)_3_]ReO_3_, and [HC(pz)_3_]ReO_3_[ReO4] and pyridine/pyridine-type ligands [(4,7-diphenyl-1,10-phen)(Br)-ReO_3_], [(4,4′-di-tert-butyl-2,2′-dipyridyl)(Cl)ReO_3_] and [(py)_2_Re(Cl)O_3_] with diphenyl ketene, isolated six novel [3 + 2] cycloaddition products. The isolated solid products were air-stable and resulted from the formation of a [3 + 2] addition of the O=Re=O motif of the rhenium oxo species across the ketene C=C double bond. Five of the six [3 + 2] cycloaddition products were structurally characterized by single-crystal X-ray diffraction and in all cases by ^13^C NMR and IR spectroscopies.

In another experimental study, Herrmann et al. (1985) reported that the stable mononuclear complex Cp* ReO_3_ can be reacted with an excess of diphenyl ketene in THF at room temperature to yield the corresponding [3 + 2] cycloaddition product. The isolated five membered [3 + 2] product was characterized by X-ray analysis.

Herrmann et al. (1994) in an experimental study, also showed that when methyltrioxorhenium (VII), MTO was coordinated with a bipyridyl ligand, the resulting octahedral complex could be reacted with an excess of diphenyl ketene in THF to yield the corresponding [3 + 2] cycloaddition product. Herrmann et al. reported that when MTO‚4-tert-butylpyridine was reacted with diphenyl ketene in the presence of an excess of 4-tert-butylpyridine base, an instantaneous [3 + 2] cycloaddition product was formed.

Deubel et al. (2001) reported a theoretical studies on the periselectivity, chemoselectivity, stereoselectivity and regioselectivity of the addition of the transition metal-oxide OsO_4_ and LReO_3_ (L = O^−^, NPH_3_, CH_3_, Cp and Cp*) to ketenes. In the reaction of LReO_3_, L = O^−^, NPH_3_, CH_3_, Cp and Cp* with ketenes, Deubel et al. found the activation barrier for the [2 + 2] addition across the C=O moiety of the ketene to be the lowest for the ligands, L = (O^−^ and NPH_3_) and the [2 + 2] addition across the C=C moiety to be the lowest for the ligands, L = CH_3_ and Cp. However, the differences between the calculated [2 + 2] activation barriers were calculated to be small.

Recently, Ahmed et al. (2015) reported density functional theory calculation at the MO6/LACVP* and B3LYP/LACVP* levels of theory on the peri-, chemo- and regio-selectivity of the addition of transition metal oxo complexes of the type ReO_3_L (L = Cl^−^, O^−^, OCH_3_, CH_3_) to substituted ketenes O=C=C (CH_3_) (X) [X = CH_3_, H, CN and Ph] with the aim of elucidating the effect of substituents on the mechanism of the reactions. They reported the [2 + 2] addition pathway across the C=C or C=O (depending on the ligand) to be the most preferred pathway in the reactions of dimethyl ketene with all the metal complexes studied. The [2 + 2] addition pathway is also the most preferred in the reactions of ReO_3_Cl with all the substituted ketenes studied except for when X = Cl where the [3 + 2] addition pathway is preferred. The order in the activation energies of the reactions of dimethyl ketenes with the metal complexes ReO_3_L with respect to changing ligand L is O^−^ < CH_3_O^−^ < Cl^−^ < CH_3_ while the order in reaction energies is CH_3_ < CH_3_O^−^ < O^−^ < Cl^−^ was reported by Ahmed et al. (2015).

In contrast to ketenes, experimental and theoretical investigations on the mechanism of addition of transition metal-oxide of the type LMO_3_ (M = Mn, Tc and Re) across the C=C double bonds of olefins has been extensively reported (Sharpless and Akashi 1975; Wiberg 1965; Aniagyei et al. 2013a, b, c; Tia and Adei 2009, 2011; Nelson et al. 1997; Corey and Noe 1997; Haller et al. 1997; Houk and Strassner 1999; Torrent et al. 1998; Del Monte et al. 1997; Tia and Adei 2011; Pietsch et al. 1998; Deubel and Frenking 1999; Gisdakis and Rösch 2001). Ketenes are related to olefins by the presence of the C=C double bonds but ketenes constitute activated double bonds due to the presence of the C=O double bond directly bonded to the C=C double bond. Therefore the chemistry of the addition of transition metal oxo complexes of the type LReO_3_ to ketenes is much expected to be the same as olefins periselectivity.

**Scheme 1:**
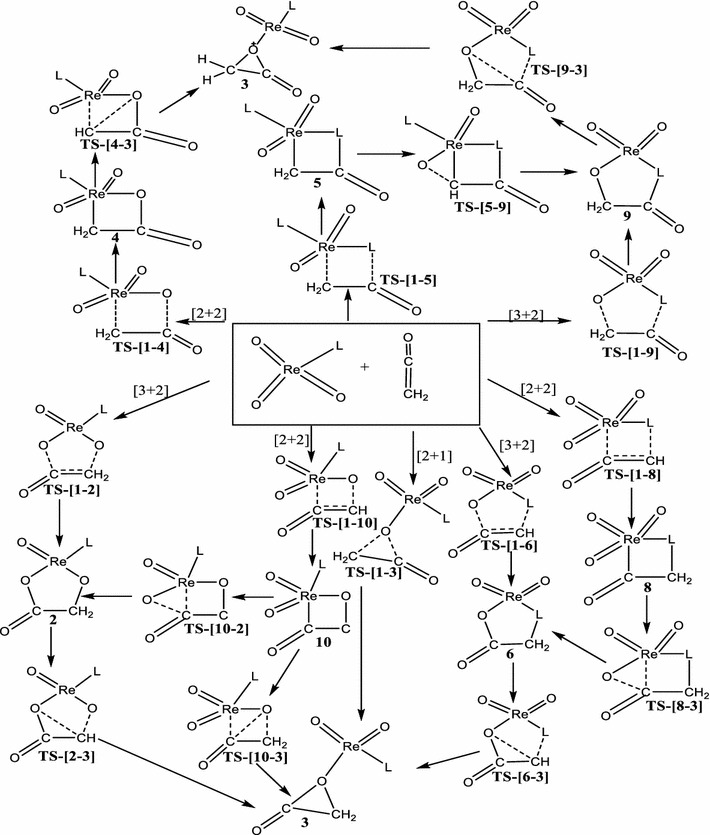
Proposed concerted addition of ReO_3_L (L = Cl^−^, CH_3_O^−^, CH_3_ and Cp) across C=C bond of C=C=O

**Scheme 2:**
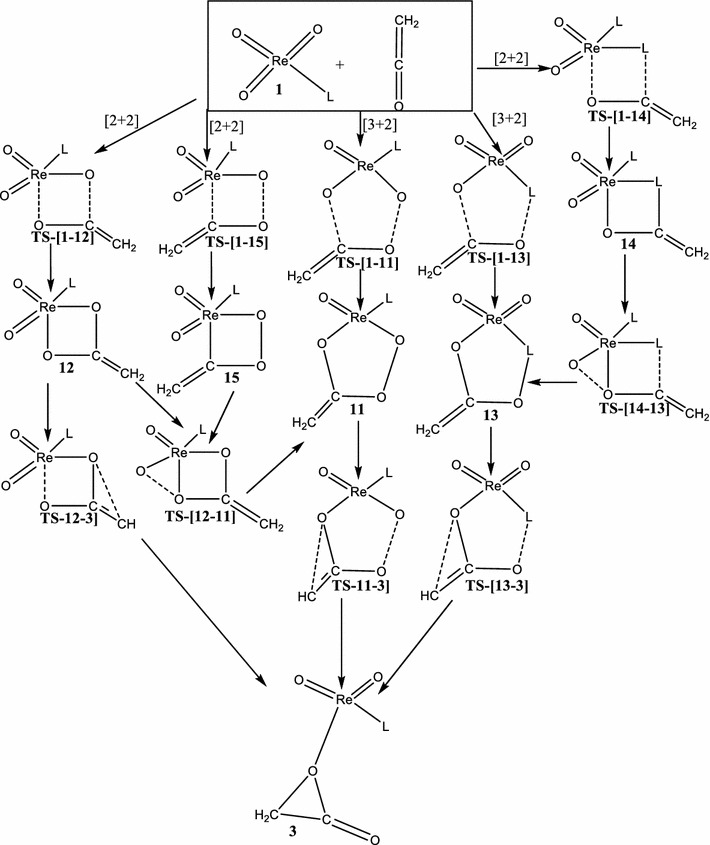
Proposed concerted addition of ReO_3_L (L = Cl^−^, CH_3_O^−^, CH_3_ and Cp) across C=O double bonds of C=C=O

**Scheme 3:**
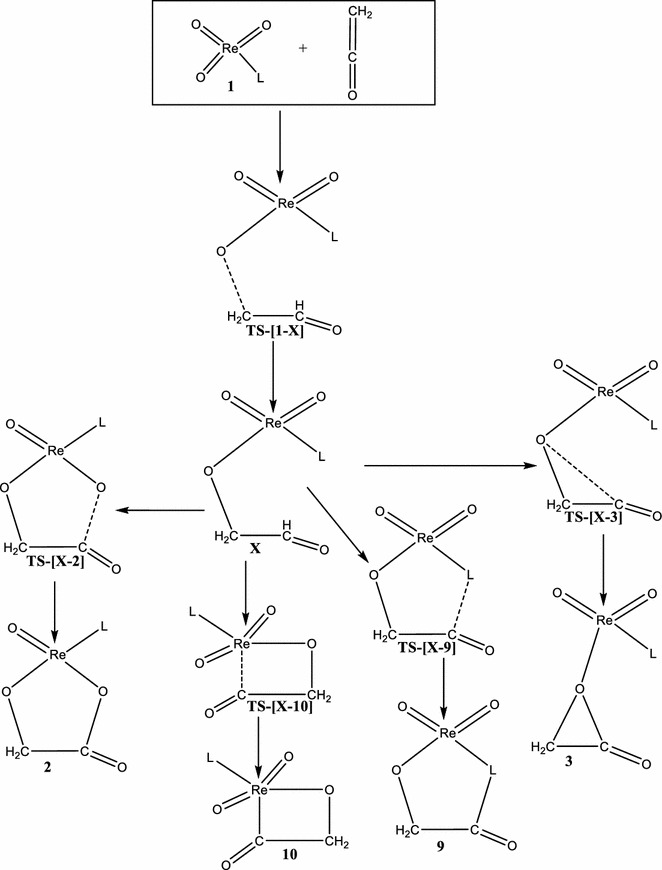
Proposed stepwise addition of ReO_3_L (L = Cl^−^, CH_3_O^−^, CH_3_, and Cp) with the C=C bond of C=C=O

**Scheme 4:**
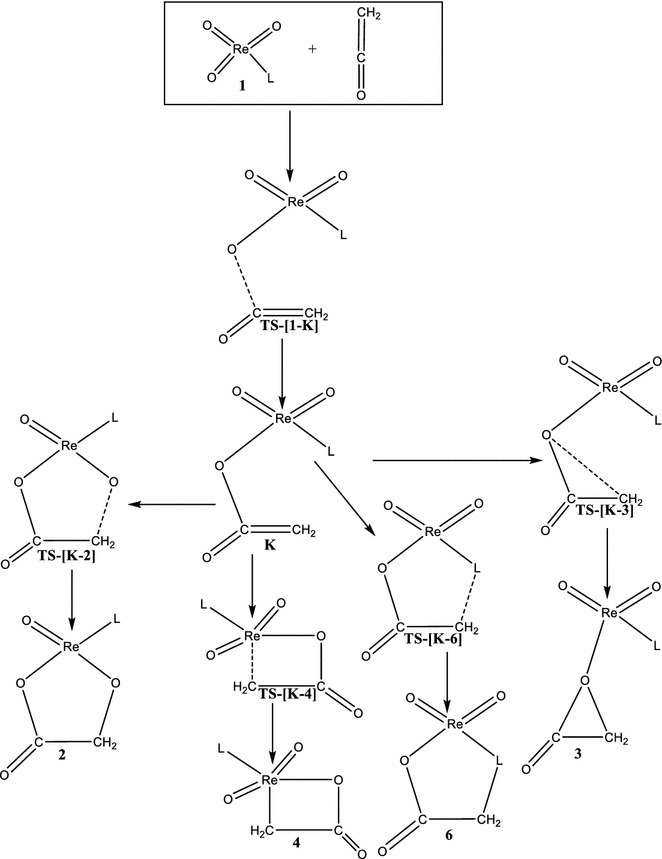
Proposed stepwise addition of ReO_3_L (L = Cl^−^, CH_3_O^−^, CH_3_, and Cp) with the C=O bond of C=C=O

In this work, the chemoselectivity and periselectivity of the addition of LReO_3_ (L = Cl, Cp, CH_3_ and OCH_3_) to ethenone is carried out by employing hybrid density functional theory at the MO6 and B3LYP/LACVP* level of theory to calculate the energetics of the concerted and stepwise [3 + 2] and [2 + 2] addition pathways. Also the possible mechanistic channels to the formation of the ethanoic acid precursor (Schemes 1, 2, 3, 4) is explored. The singlet and triplet spin states have been considered in this work. The addition of the metal oxides LReO_3_ across the C=O moiety of the ethenone leading to the [2 + 2] products such as metalla-2-oxolanes and metalla-2,3-dioxolane and also the [3 + 2] products such as metalla-2,3,5-trioxolane which were not explored in the previous works of Deubel et al. (2001) and Ahmed et al. (2015) are fully explored in this studies. Also the nucleophilic attack of both the carbonyl carbon and the adjacent carbon atom on an oxo ligand of the metal oxide is explored in this studies which was partially explored in the work of Ahmed et al. (2015) and not explored in the work of Deubel et al. (2001). Knowledge of the mechanistic details of these kinds of reactions might open new methods for efficient activation of double bonds by readily accessible transition metal-oxides, beyond the common methods of *cis*-hydroxylation using metal-oxides.

## Details of calculation

We performed all the computations with the Spartan (2010) computational chemistry package developed by Wavefunction, Inc., versions 2008V1.2.0 and 2010V1.2.0, using the Becke-three-parameter Lee–Yang–Parr (B3LYP) hybrid exchange–correlational functional and the MO6 hybrid functional. The B3LYP functional, a Hartree–Fock DFT hybrid functional, is made up of the exchange–correlation energy from the local spin-density approximation (LSDA) method, 20 % of the difference between the Hartree–Fock exchange energy (Kohn–Sham exchange energy) and the LSDA exchange energy, 72 % of the Becke exchange potential (which includes the 1988 correction) (Becke 1988, 1996), 81 % of the Lee–Yang–Parr correlation potential (Lee et al. 1988) and 19 % of the Vosko–Wilk–Nusair potential (Vosko et al. 1980) and is one of the most widely used exchange-correlation functionals in organometallic chemistry. The MO6 functional (Zhao and Truhlar 2008) is a global hybrid meta-generalized gradient approximation (meta-GGA) with 27 % of Hartree–Fock exchange, leading to a well-balanced functional for overall good performance for chemistry. It has thus been recommended for application in organometallic and inorganometallic chemistry (Peverati and Truhlar 2014). The atoms hydrogen-chlorine were described with the 6-31G (d) basis set while the metal Re was described with the LANL2DZ basis set (Dunning and Hay 1976; Hay and Wadt 1985a, 1985b; Wadt and Hay 1985; Roy et al. 2008). The MO6/LANL2DZ and B3LYP/LANL2DZ are the two most popular DFT levels of theory for organometallic and inorganic chemistry to date. The latest review article on DFT methods for computational studies of synthetically relevant homogeneous organometallic catalysis (Sperger et al. 2015) indicates that between the period 2009–2014 geometry optimization calculations are dominated by the LANL2DZ as ECP for the transition metal. Table 1d of that review shows that for the period 2013–2014, 51 % of all reviewed studies employed the LAN2DZ basis set for geometry optimization. The same review indicates that currently, the first choice of functional for energy calculations of TM systems is M06, followed by B3LYP, DFT-D3, and M06L, whereas the choice of basis set for the description of the transition metal is dominated by LANL2DZ and SDD (the Stuttgart–Dresden ECP with double zeta basis set).

Spartan uses a graphical model builder for input preparation. Molecules were constructed and minimized interactively using an appropriate molecular mechanics force field. All structural optimizations were done without symmetry restrictions. Normal mode analysis was performed to verify the nature of the stationary points located. Minima, representing reactants, intermediates and products were shown to have no imaginary frequencies.

Guess structures for transition state calculations were obtained by first constraining specific bonds along the reaction coordinates at fixed lengths while the remaining internal coordinates were fully optimized. This procedure gives an approximate transition state guess which is then submitted for transition state calculation using the standard transition state optimization procedure in Spartan. All transition state structures were subjected to full normal mode analyses to ensure that they have a Hessian matrix with a single negative eigen-value, characterized by an imaginary vibrational frequency along the reaction coordinate. An intrinsic reaction coordinate (IRC) calculation was carried out to ensure that transition states smoothly connect reactants and products.

## Results and discussion

### Reaction of ReO_3_Cl with ethenone

**Fig. 1:**
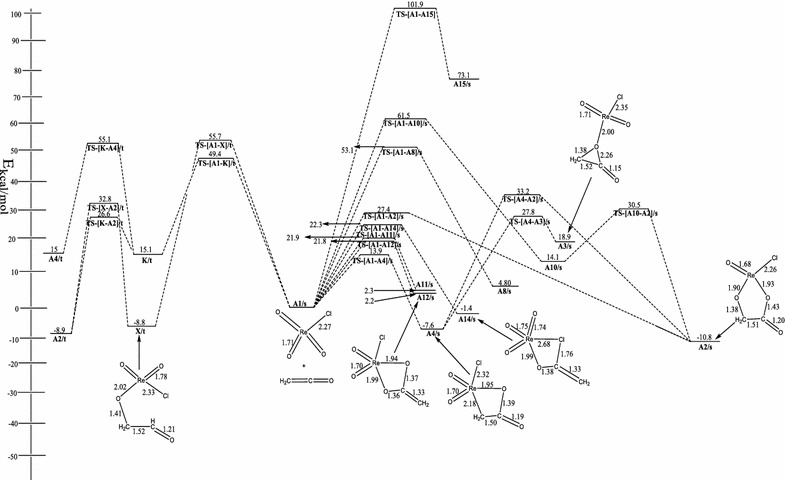
Energetics of the reaction of ReO_3_Cl with ethenone on the singlet and triplet PES at B3LYP/LACVP*

Figure 1 shows the relative energies of the main stationary points (reactants, transition states, intermediates and products) involved in the reaction between ReO_3_Cl and ethylene as well as some of the optimized structures.

The singlet ReO_3_Cl has all the Re–O bonds equal at 1.70 Å and the Re–Cl bond at 2.27 Å. The singlet structure is 62.7 kcal/mol more stable than the triplet structure.

On the singlet potential energy surface (PES), the concerted [3 + 2] addition of the C=C bond of ethenone (C=C=O) across the O=Re=O bond of singlet ReO_3_Cl to form metalla-2,5-dioxolane-3-one, **A2/s** (Fig. 1) has an activation barrier of 27.4 kcal/mol and an exothermicity of 10.8 kcal/mol through the singlet transition state **TS-[A1–A2]/s**. At the M06 level of theory, the activation barrier and exothermicity are calculated to be 28.2 and 17.9 kcal/mol.

On the triplet PES, the formation of **A2/t** proceed in a stepwise manner as depicted in Schemes 3 and 4. In one route, the carbon atom adjacent the carbonyl carbon of the C=C=O attacks an oxo ligand of ReO_3_Cl to form the intermediate **X/t** through a triplet transition state **TS-[A1–X]/t**. The activation and reaction energy for the formation of this intermediate are 55.7 and −8.8 kcal/mol respectively. The triplet intermediate **X/t**, then re-arranges to **A2/t** through a triplet transition state **TS-[X–A2]/t** with an activation barrier of 41.6 kcal/mol and reaction energy of −0.1 kcal/mol.

Alternatively, **A2/t** could be formed by direct attack of the carbonyl carbon of ethenone (C=C=O) on an oxo ligand of ReO_3_Cl to form the intermediate **K/t** through a triplet transition state **TS-[A1–K]/t**. The activation and reaction energy for the formation of this triplet intermediate are 49.4 and 15.1 kcal/mol respectively. The triplet intermediate **K/t**, then re-arranges to **A2/t** through a triplet transition state **TS-[K–A2]/t** with an activation barrier of 11.50 kcal/mol and reaction energy of 24.0 kcal/mol.

Though both stepwise routes leads to the formation of **A2/t**, the attack of the carbonyl carbon on the oxo ligand of ReO_3_Cl is the favorable and leads to the formation of **A2/t**. The species **A2/s** has been found to be 10.7 kcal/mol more stable than the triplet counterpart **A2/t**. The rearrangement of the metalla-2,5-dioxolane-3-one intermediate, **A2** to the acetic acid precursor on both the singlet and triplet PES could not located.

The formation of metalla-2-oxetane-3-one, **A4/s** (Fig. 1) resulted from the direct [2 + 2] addition of the C=C bond of ethenone across the Re=O bond of singlet ReO_3_Cl. The activation barrier and exothermicity of the process are 13.9 and 7.6 kcal/mol respectively. At the MO6 level, activation and reaction energies are 9.7 and 14.4 kcal/mol respectively. On the triplet surface, the formation of **A4/t** follows a stepwise pathway. The activation and reaction energies involved in the formation of **A4/t** are 40.1 and −0.1 kcal/mol respectively. Species **A2/s** could be formed from **A4/s** through the singlet transition state **TS-[A4–A2]/s**. The activation barrier for the rearrangement process is 40.8 kcal/mol. The formation of the acetic acid precursor **A3/s** (Fig. 1) could be achieved through the cyclization of the metalla-2-oxetane-3-one intermediate, **A4/s**. The activation barrier and reaction energy of the process are 35.4 kcal/mol and endothermicity of 26.5 kcal/mol.

The calculated activation and reaction energies for the [2 + 2] addition of the C=C moiety of ketene across the Re=O functionality of ReO_3_Cl is about 4 and 6.8 kcal/mol lower at the M06 level than at the B3LYP level of theory. Since the difference in energy barrier for the [3 + 2] addition pathways at the B3LYP and MO6 level of theory is similar, the remaining calculations were performed at the B3LYP/LACVP* level of theory. The MO6 results do not change the preferred addition pathway.

The formation of metalla-2-oxetane-4-one, **A10/s** (**10** in Scheme 1) by the direct [2 + 2] addition of the C=C bond of ethenone across the Re=O bond of singlet ReO_3_Cl through the singlet transition state **TS-[A1–A10]/s** has an activation barrier and exothermicity of the process are 61.5 and 14.1 kcal/mol respectively. An exhaustive attempt to locate the triplet species and the acetic acid precursor through the rearrangement route proved futile. **A10/s** could rearrange to **A2/s** through the singlet transition state **TS-[A10–A2]/s** to form the five membered metallacycle **A2/s**. The activation barrier for the rearrangement process is 16.4 kcal/mol.

The concerted [2 + 2] addition of the C=C functionality of C=C=O across the Re–Cl bond of singlet ReO_3_Cl leads to the formation of metalla-2-chloro-4-one, **A8/s** through the singlet transition state **TS-[A1–A8]/s**. The activation barrier and endothermicity for the process are 53.1 and 4.80 kcal/mol. No triplet counterpart of **A8/s** was found on the PES. No triplet counterpart of **A8/s** and acetic acid precursor rearrangement pathways was located on both the singlet and triplet PES.

On the singlet PES, the concerted [3 + 2] addition of the C=O bond of ethenone (C=C=O) across the O=Re=O bond of singlet ReO_3_Cl to form metalla-2,3,5-trioxolane, **A11/s** (**11** in Scheme 2) has an activation barrier of 21.9 kcal/mol and an endothermicity of 2.3 kcal/mol through the singlet transition state **TS-[A1–A11]/s**. No triplet species was obtained. Also an attempt to locate an acetic acid precursor through the rearrangement of **A11/s** intermediate did not yield any results. The formation of metalla-2,4-dioxolane **A12/s** (Fig. 1) on the singlet PES by the direct [2 + 2] addition of the C=O moiety of C=C=O across the Re=O bond of singlet ReO_3_Cl has activation and reaction energies of 21.8 and 2.2 kcal/mol respectively. No triplet counterpart of **A12/s** was located on the surface.

Also the concerted [2 + 2] addition of the C=O moiety of C=C=O across the Re–Cl bond of singlet ReO_3_Cl leads to the formation of metalla-2-chloro-oxolane, **A14/s** through the singlet transition state **TS-[A1–A14]/s**. The activation barrier and exothermicity for the formation process are 22.3 and 1.4 kcal/mol. An exhaustive search on the surface for the triplet counterpart of **A14/s** and acetic acid precursor rearrangement routes did not yield any results. The formation of metalla-2,3-dioxolane **A15/s** (**15** in Scheme 2) on the singlet PES by the direct [2 + 2] addition of the C=O moiety of C=C=O across the Re=O bond of singlet ReO_3_Cl has activation and reaction energies of 101.9 and 73.1 kcal/mol respectively. No triplet counterpart of **A15/s** and acetic acid precursor was located on the surface.

### Reaction of ReO_3_CH_3_ with ethenone

**Fig. 2:**
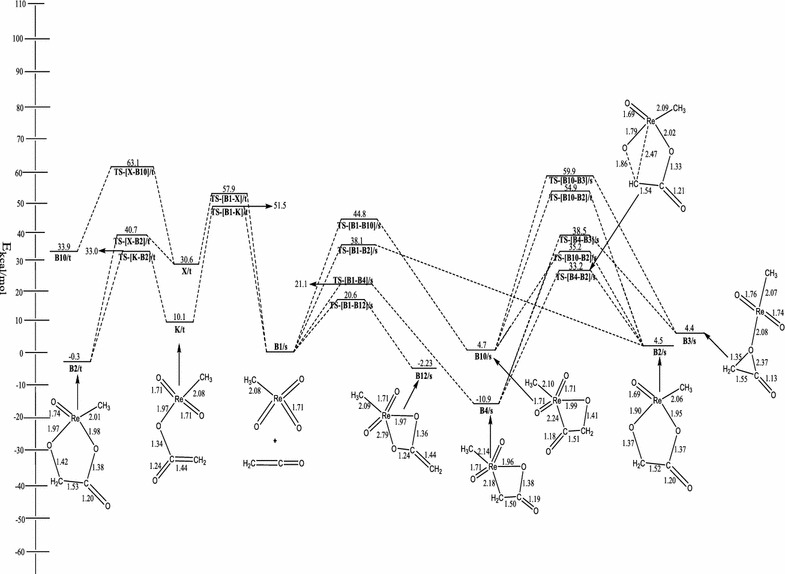
Energetics of the reaction of ReO_3_CH_3_ with ethenone on the singlet and triplet PES at B3LYP/LACVP*

The optimized geometries and the energy profile of the reaction of ReO_3_ (CH_3_) with ethenone is shown in Fig. 2. The singlet reactant is calculated to be 68.0 kcal/mol more stable than the triplet reactant.

On the singlet PES, the formation of metalla-2,5-dioxolane-3-ones, species **B2/s** by the concerted [3 + 2] addition of the C=C functionality of ethenone across the O=Re=O bonds of singlet ReO_3_ (CH_3_) has activation barrier of 38.1 kcal/mol and endothermicity of 4.5 kcal/mol. Deubel et al. (2001) calculated the activation barrier and reaction energy for this route to be 44.5 and 16.3 kcal/mol respectively at the B3LYP/III+//B3LYP/II level with relativistic small-core ECPs and a (441/2111/21) valence-basis set.

On the triplet PES, the formation of **B2** which was not reported in the work of Deubel and co-workers (Del Monte et al. 1997) proceeds in a stepwise manner as depicted in Schemes 3 and 4. In one route (Scheme 3), the carbon atom adjacent the carbonyl carbon of the C=C=O attacks an oxo ligand of ReO_3_CH_3_ to form the intermediate **X/t** through a triplet transition state **TS-[B1–X]/t**. The activation and reaction energy for the formation of this intermediate are 57.9 and 30.6 kcal/mol respectively. **B2/t** could then be formed from the triplet intermediate **X/t**, through the triplet transition state **TS-[X–B2]/t** with an activation barrier of 10.1 kcal/mol and exothermicity of −30.9 kcal/mol. Also, **B2/t** could be formed by direct attack of the carbonyl carbon of ethenone (C=C=O) on an oxo ligand of ReO_3_Cl (Scheme 4) to form the organometallic intermediate **K/t** through a triplet transition state **TS-[B1–K]/t**. The activation and reaction energy for the formation of this triplet intermediate are 51.5 and 10.1 kcal/mol respectively. The triplet intermediate **K/t**, then re-arranges to **B2/t** through a triplet transition state **TS-[K–B2]/t** with an activation barrier of 22.90 kcal/mol and reaction energy of −10.4 kcal/mol. The triplet species **B2/t** is 5.9 kcal/mol more stable than **B2/s**. The acetic acid precursor rearrangement could be located on the reaction surfaces.

On the singlet PES, the formation of the metalla-2-oxetane-3-one, **B4/s** (Fig. 2) is concerted. The activation barrier and exothermicity of the process are 21.1 and 10.9 kcal/mol. Deubel and co-workers (Del Monte et al. 1997) reported values of 20.2 and −6.3 kcal/mol for the activation and reaction energies at the B3LYP/III+//B3LYP/II level with relativistic small-core ECPs and a (441/2111/21) valence-basis set.

Metalla-2,5-dioxolane-3-one, **B2/s** could be formed from **B4/s** through the singlet transition state **TS-[B4–B2]/s**. The activation barrier for this route is 44.1 kcal/mol. Deubel and co-workers (Del Monte et al. 1997) reported the activation barrier for the re-arrangement route to be 46.5 kcal/mol at the B3LYP/III+//B3LYP/II level with relativistic small-core ECPs and a (441/2111/21) valence-basis set. No triplet species and acetic acid precursor could be located on the reaction surfaces. The acetic acid precursor **B3/s** can be formed from the **B4/s** intermediate through the rearrangement transition state **TS-[B4–B3]/s**. The activation barrier and reaction energy for the formation process is 49.4 kcal/mol respectively.

On the singlet PES, the formation of metalla-2-oxetane-4-one, **B10/s** (Fig. 2) is concerted. The activation barrier and endothermicity of the process are 44.8 and 4.7 kcal/mol. On the triplet surface, the carbon atom adjacent the carbonyl carbon of the C=C=O attacks an oxo ligand of ReO_3_CH_3_ to form the intermediate **X/t** through a triplet transition state **TS-[B1–X]/t**. The activation and reaction energy for the formation of this intermediate are 57.9 and 30.6 kcal/mol respectively. **B10/t** could then be formed from the triplet intermediate **X/t**, through the triplet transition state **TS-[X–B10]/t** with an activation barrier of 32.5 kcal/mol and endothermicity of 3.3 kcal/mol. **B2/s** could also be formed from **B10/s** through the singlet transition state **TS-[B10–B2]/s**. The activation barrier for this route is 30.5 kcal/mol. On the triplet surface, the activation barrier is 50.2 kcal/mol. The acetic acid precursor **B3/s** is formed from **B10/s** through the transition state **TS-[B10–B3]/s**. The activation barrier for the process is 55.2 kcal/mol.

The formation of metalla-2,4-dioxolane, **B12/s** on the singlet PES by the direct [2 + 2] addition of C=O functionality of ethenone across the Re=O bond of singlet ReO_3_CH_3_ has activation barrier of 20.6 kcal/mol and exothermicity of 2.2 kcal/mol. Deubel et al. (2001) reported values of 23.3 and 0.5 kcal/mol for the activation and reaction energy respectively at the B3LYP/III+//B3LYP/II level with relativistic small-core ECPs and a (441/2111/21) valence-basis set.

On the triplet surface and not explored in Deubel et al. (2001) work, the concerted addition leading to the formation of **B12/t** has activation barrier of 49.1 kcal/mol and endothermicity of 9.9 kcal/mol. The singlet species **B12/s** has been found to be 7.7 kcal/mol more stable than **B12/t**.

### Reaction of ReO_3_(OCH_3_) with ethenone

**Fig. 3:**
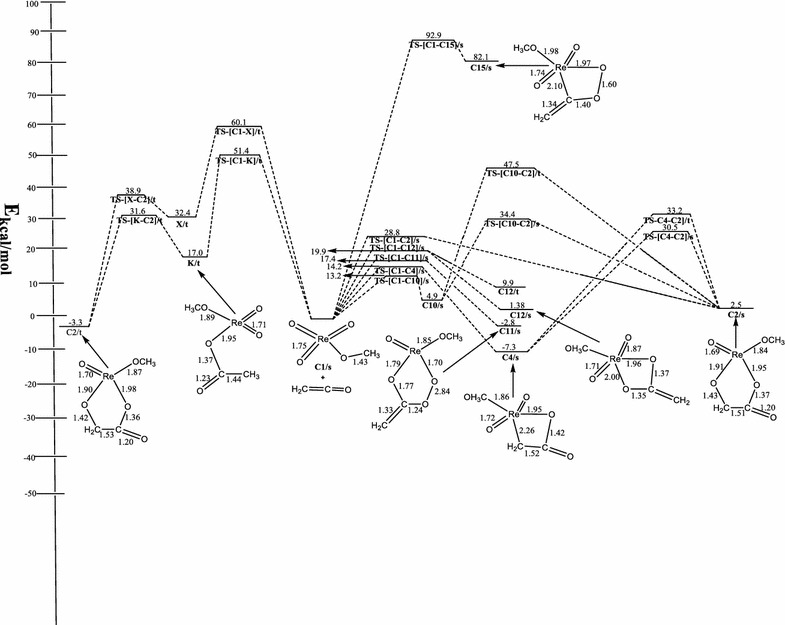
Energetics of the reaction of ReO_3_OCH_3_ with ethenone on the singlet and triplet PES at B3LYP/LACVP*

Figure 3 shows the optimized geometries and relative energies of the main stationary points involved in the reaction between ReO_3_(OCH_3_) and ethenone. The singlet reactant **C1/s** is 63.8 kcal/mol more stable than the triplet **C1/t**.

On the singlet PES, the concerted [3 + 2] addition of the C=C bond of ethenone (C=C=O) across the O=Re=O bond of singlet ReO_3_OCH_3_ to form metalla-2,5-dioxolane-3-one, **C2/s** (Fig. 3) has an activation barrier of 28.8 kcal/mol and an endothermicity of 2.5 kcal/mol through the singlet transition state **TS-[C1–C2]/s**. On the triplet PES, the formation of **C2/t** proceed in a stepwise manner as depicted in Schemes 3 and 4. In one route, the carbon atom adjacent the carbonyl carbon of the C=C=O attacks an oxo ligand of ReO_3_OCH_3_ to form the intermediate **X/t** through a triplet transition state **TS-[C1-X]/t**. The activation and reaction energy for the formation of this intermediate are 60.1 and 32.4 kcal/mol respectively. The triplet intermediate **X/t**, then re-arranges to **C2/t** through a triplet transition state **TS-[X–C2]/t** with an activation barrier of 6.5 kcal/mol and reaction energy of −35.7 kcal/mol.

Alternatively, **C2/t** could be formed by direct attack of the carbonyl carbon of ethenone (C=C=O) on an oxo ligand of ReO_3_Cl to form the intermediate **K/t** through a triplet transition state **TS-[C1–K]/t**. The activation and reaction energy for the formation of this triplet intermediate are 51.4 and 17 kcal/mol respectively. The triplet intermediate **K/t**, then re-arranges to **C2/t** through a triplet transition state **TS-[K–C2]/t** with an activation barrier of 14.6 kcal/mol and reaction energy of −20.3 kcal/mol. Though both stepwise routes leads to the formation of **C2/t**, the attack of the carbonyl carbon on the oxo ligand of ReO_3_Cl is the favorable and leads to the formation of **C2/t**. The species **C2/t** has been found to be 0.8 kcal/mol more stable than **C2/s**.

The rearrangement of the metalla-2,5-dioxolane-3-one intermediate, **C2** to the acetic acid precursor on both the singlet and triplet PES could not located.

The formation of metalla-2-oxetane-3-one, **C4/s** (Fig. 3) resulted from the direct [2 + 2] addition of the C=C bond of ethenone across the Re=O bond of singlet ReO_3_OCH_3_. The activation barrier and exothermicity of the process are 14.2 and 7.3 kcal/mol respectively. Species **C2/s** could be formed from **C4/s** through the transition state **TS-[C4–C2]/s**. The activation barrier for the rearrangement process is 37.8 kcal/mol. On the triplet surface, the activation barrier for the rearrangement process is 40.5 kcal/mol. A search for the formation of the acetic acid precursor, **3** whether through the direct addition pathway or rearrangement of the metalla-2-oxetane-3-one intermediate both on the singlet and triplet surfaces proved unsuccessful.

The formation of metalla-2-oxetane-4-one, **C10/s** (Fig. 3) by the direct [2 + 2] addition of the C=C bond of ethenone across the Re=O bond of singlet ReO_3_OCH_3_ through the singlet transition state **TS-[C1–C10]/s** has an activation barrier and endothermicity of the process are 13.2 and 4.9 kcal/mol respectively. **C10/s** could rearrange to **C2/s** through the transition state **TS-[C10–C2]/s** to form the five membered metallacycle **C2/s**. The activation barrier for the rearrangement process is 29.5 kcal/mol. On the triplet surface, the activation barrier for the rearrangement process is 42.6 kcal/mol. An exhaustive attempt to locate the triplet counterpart of **C2/s** and the acetic acid precursor through the direct and rearrangement route proved futile.

The concerted [3 + 2] addition of the C=O bond of ethenone(C=C=O) across the O=Re=O bond of singlet ReO_3_OCH_3_ to form metalla-2,3,5-trioxolane, **C11/s** has an activation barrier of 17.4 kcal/mol and an exothermicity of 2.8 kcal/mol through the transition state **TS-[C1–C11]/s**. No triplet species was obtained. Also an attempt to locate an acetic acid precursor through the rearrangement of **C11/s** intermediate did not yield any results. The formation of metalla-2,4-dioxolane **C12/s** (Fig. 3) on the singlet PES by the direct [2 + 2] addition of the C=O moiety of C=C=O across the Re=O bond of singlet ReO_3_OCH_3_ has activation and reaction energies of 19.9 and 1.38 kcal/mol respectively. The triplet counterpart of **C12/s** was computed to be 9.9 kcal/mol. No acetic acid precursor is located on both the singlet and triplet PES explored.

The formation of metalla-2,3-dioxolane **C15/s** on the singlet PES by the direct [2 + 2] addition of the C=O moiety of C=C=O across the Re=O bond of singlet ReO_3_OCH_3_ has activation and reaction energies of 92.9 and 82.1 kcal/mol respectively. No triplet counterpart of **C15/s** and acetic acid precursor were located on the surface.

### Reaction of ReO_3_Cp with ethenone

**Fig. 4:**
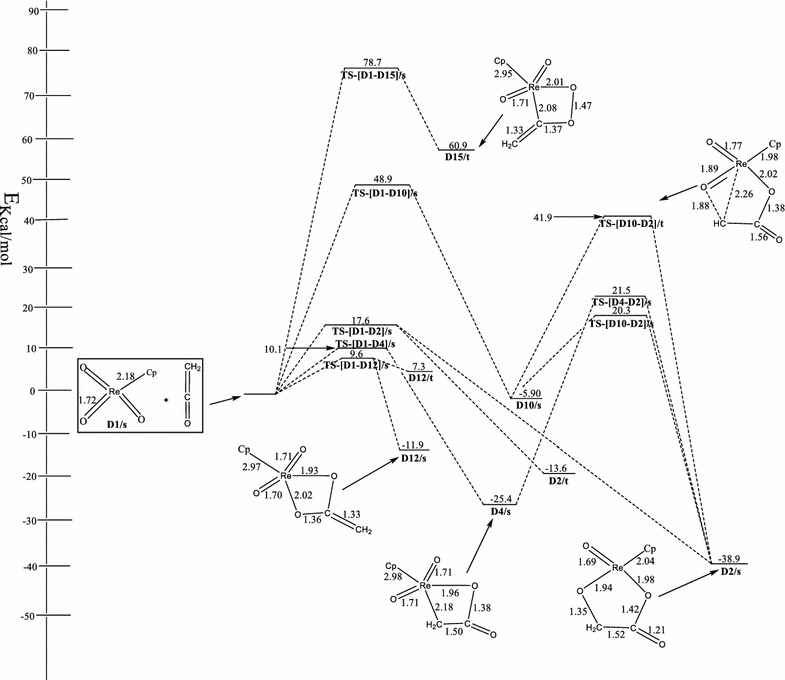
Energetics of the reaction of ReO_3_OCp with ethenone on the singlet and triplet PES at B3LYP/LACVP*

Figure 4 shows the optimized geometries and relative energies of the main stationary points involved in the reaction between ReO_3_Cp and ethylene. In the singlet reactant, the cyclopentadienyl ligand (Cp) is bonded to the metal center in a η^5^-fashion i.e. Re-C (Cp) = 2.49, 2.51, 2.52, 2.49 and 2.46 Å, in agreement with work of Deubel and Frenking (Wiberg 1965; Pietsch et al. 1998). No triplet species could be located for the reactant.

On the singlet PES, the formation of metalla-2,5-dioxolane-3-ones, species **D2/s** by the concerted [3 + 2] addition of the C=C functionality of ethenone across the O=Re=O bonds of singlet ReO_3_Cp has activation barrier of 17.6 kcal/mol and exothermicity of 38.9 kcal/mol. Deubel et al. (2001) reported the activation barrier and reaction energy for this route to be 24.8 and −29.2 kcal/mol respectively at the B3LYP/III+//B3LYP/II level with relativistic small-core ECPs and a (441/2111/21) valence-basis set.

An attempt to locate the acetic acid precursor through the direct addition pathway and the rearrangement of species **D2/s** proved unsuccessful.

The formation of the metalla-2-oxetane-3-one, **D4/s** (Fig. 4) is concerted. The activation barrier and exothermicity of the process are 10.1 and 25.4 kcal/mol. Deubel et al. (2001) reported values of 11.8 and −20.2 kcal/mol for the activation and reaction energies at the B3LYP/III+//B3LYP/II level with relativistic small-core ECPs and a (441/2111/21) valence-basis set. Metalla-2,5-dioxolane-3-one, **D2/s** could be formed from **D4/s** through the singlet transition state **TS-[D4–D2]/s**. The activation barrier for this route is 46.4 kcal/mol. No triplet species and acetic acid precursor could be located on the surfaces.

Also, the formation of metalla-2-oxetane-4-one, **D10/s** on the singlet surface is concerted. The activation barrier and exothermicity of the process are 48.9 and 5.90 kcal/mol. **D10/s** rearranges to **D2/s** through the transition state **TS-[D10–D2]/s**. The activation barrier for this route is 26.2 kcal/mol. On the triplet surface, the activation barrier is 47.8 kcal/mol. An attempt to locate the acetic acid precursor either from the direct addition pathway or the rearrangement of **D10/s** proved futile.

The formation of metalla-2,4-dioxolane, **D12/s** on the singlet PES by the direct [2 + 2] addition of C=O functionality of ethenone across the Re=O bond of singlet ReO_3_Cp has activation barrier of 9.6 kcal/mol and exothermicity of 11.9 kcal/mol. Deubel et al. (2001) reported values of 12.9 and −5.3 kcal/mol for the activation and reaction energy respectively at the B3LYP/III+//B3LYP/II level with relativistic small-core ECPs and a (441/2111/21) valence-basis set. On the triplet surface not explored in Deubel et al. (2001) work, the concerted addition leading to the formation of **D12/t** has a reaction energy of 7.3 kcal/mol. Species **D12/s** has been found to be 19.2 kcal/mol more stable than **D12/t**. The rearrangement of the singlet and triplet **D2** intermediate to the acetic acid precursor proved unsuccessful.

The formation of metalla-2,3-dioxolane **D15/s** on the singlet PES by the direct [2 + 2] addition of the C=O moiety of C=C=O across the Re=O bond of singlet ReO_3_Cp has activation and reaction energies of 78.7 and 60.9 kcal/mol respectively. No triplet counterpart of **D15/s** and acetic acid precursor were located on the surfaces.

## Conclusions

In the reaction of LReO_3_ (L = Cl^−^, OCH_3_, CH_3_ and Cp) with ethenone at the B3LYP/LANL2DZ level of theory, the concerted [2 + 2] addition of the metal oxide across the C=C double bond to form metalla-2-oxetane-3-one, **4** and C=O functionality of ethenone also leading to the formation of metalla-2,4-dioxolane, **12** is the most kinetically favored over the metalla-2,5-dioxolane-3-one, **2** from the direct [3 + 2] addition pathway. The trends in activation and reaction energies for the formation of metalla-2-oxetane-3-one, **4** and metalla-2,4-dioxolane, **12** are Cp < Cl^−^ < OCH_3_ < CH_3_ and Cp < OCH_3_ < CH_3_ < Cl^−^ and reaction energies are Cp < OCH_3_ < Cl^−^ < CH_3_ and Cp < CH_3_ < OCH_3_ < Cl CH_3_. The concerted [3 + 2] addition of the metal oxide across the C=C double bond to form metalla-2,5-dioxolane-3-one, **2** is thermodynamically the most favored for the ligand (L = Cp). The direct [2 + 2] addition pathways leading to the formations of metalla-2-oxetane-3-one, **4** and metalla-2-oxetane-4-one, **10** is thermodynamically the most favored for the ligand (L = OCH_3_ and Cl^−^). However, the difference between the calculated [2 + 2] activation barriers for the addition of the metal oxide LReO_3_ across the C=C and C=O functionalities of ethenone leading to the formations of metalla-2-oxetane-3-one, **4** and metalla-2,4-dioxolane, **12** are small except for the case of L = Cl^−^ and OCH_3_. The rearrangement of the metalla-2-oxetane-4-one **10** to metalla-2,5-dioxolane-3-one **2**, even though feasible, are unfavorable due to high activation energies of their rate determining steps. In the case of the rearrangement of the metalla-2-oxetane-3-one, **4** to metalla-2,5-dioxolane-3-one, the activation barriers involved for the second steps are high and not competitive with the activation barrier involved in the direct [3 + 2] addition pathway leading to the formation of metalla-2,5-dioxolane-3-one **2**. For the rearrangement of the four membered metallacycle, metalla-2-oxetane-3-one, **4** and metalla-2-oxetane-4-one, **10** to the five membered metallacycle, metalla-2,5-dioxolane-3-one **2**, the trends in activation barriers is found to follow the order OCH_3_ < Cl^−^ < CH_3_ < Cp and Cl^−^ < Cp < OCH_3_ < CH_3_. The trends in the activation energies for the most favorable [2 + 2] addition pathways for the LReO_3_-ethenone system is CH_3_ > CH_3_O^−^ > Cl^−^ > Cp. For the analogous ethylene–LReO_3_ system, the trends in activation and reaction energies for the most favorable [3 + 2] addition pathway is CH_3_ > CH_3_O^−^ > Cl^−^ > Cp [10]. Even though the most favored pathway in the ethylene-LReO_3_ system is the [3 + 2] addition pathway and that on the LReO_3_-ethenone is the [2 + 2] addition pathway, the trends in the activation energies for both pathways are the same, i.e. CH_3_ > CH_3_O^−^ > Cl^−^ > Cp. However, the trends in reaction energies are quite different due to different product stabilities.

The formation of the acetic acid precursor **3** through the direct addition pathways was unsuccessful for all the ligands studied. The activation barriers for the formation of the acetic acid precursor through the cyclization of the metalla-2-oxetane-3-one, **4** is only possible for the ligands L = Cl^−^, CH_3_ whiles for the cyclization of metalla-2-oxetane-4-one, **10** to the acetic acid precursor is only possible for the ligand CH_3_. The formation of the metalla-2,3,5-trioxolane, **11** from the direct [3 + 2] addition of ReO_3_L (L = Cl^−^) to the C=O bond of ethenone is competitive to the direct [2 + 2] addition to form metalla-2,4-dioxolane, **12** when L = Cl^−^ and also kinetically most favored over the direct [3 + 2] addition of ReO_3_L (L = Cl^−^) to form metalla-2,5-dioxolane-3-one, **2**. The formation of metalla-2,3-dioxolane, **15** for the ligand, (L = Cl^−^, Cp and CH_3_O^−^) studied are uncompetitive in terms of higher activation and reaction energies. The trends in activation and reaction energies are found to follow the order Cp < CH_3_O^−^ < Cl and Cp < Cl < CH_3_O^−^. Although there are spin-crossover reaction observed for the ligands (L = Cl^−^, CH_3_ and CH_3_O^−^), the reactions occurring on the single surfaces have been found to occur with lower energies than their spin-crossover counterparts.
